# Smart window coating based on F-TiO_2_-K_x_WO_3_ nanocomposites with heat shielding, ultraviolet isolating, hydrophilic and photocatalytic performance

**DOI:** 10.1038/srep27373

**Published:** 2016-06-06

**Authors:** Tongyao Liu, Bin Liu, Jing Wang, Linfen Yang, Xinlong Ma, Hao Li, Yihong Zhang, Shu Yin, Tsugio Sato, Tohru Sekino, Yuhua Wang

**Affiliations:** 1 Department of Materials Science, School of Physical Science and Technology, Lanzhou University, Lanzhou, 730000, China; 2 Institute of Multidisciplinary Research for Advanced Materials, Tohoku University, 2-1-1 Katahira, Aoba-ku, Sendai, Japan; 3 The Institute of Scientific and Industrial Research, Osaka University, Japan

## Abstract

A series of smart window coated multifunctional NIR shielding-photocatalytic films were fabricated successfully through K_x_WO_3_ and F-TiO_2_ in a low-cost and environmentally friendly process. Based on the synergistic effect of K_x_WO_3_ and F-TiO_2_, the optimal proportion of K_x_WO_3_ to F-TiO_2_ was investigated and the FT/2KWO nanocomposite film exhibited strong near-infrared, ultraviolet light shielding ability, good visible light transmittance, high photocatalytic activity and excellent hydrophilic capacity. This film exhibited better thermal insulation capacity than ITO and higher photocatalytic activity than P25. Meanwhile, the excellent stability of this film was examined by the cycle photocatalytic degradation and thermal insulation experiments. Overall, this work is expected to provide a possibility in integrating K_x_WO_3_ with F-TiO_2_, so as to obtain a multifunctional NIR shielding-photocatalytic nanocomposite film in helping solve the energy crisis and deteriorating environmental issues.

Today, energy crisis and deteriorating environmental issues are posing serious threats to the sustainable development of human society. Thus, an increasing number of researches have focused on utilizing green techniques to deal with these aforementioned concerns in the past years^1^,^2^,^3^,^4^,^5^. As a critical component of buildings, windows can lose energy easily. Therefore, energy-efficient window coatings are considered to be the important step for reducing heat transfer between the indoor and outside environments. As a novel near-infrared (NIR) shielding material, the tungsten bronze (M_x_WO_3_), which is WO_3_ doped with monovalent ions such as K^+^, Na, NH_4_^+^ and others, has attracted great attention in recent years^6^,^7^. Compared with some transparent thermal insulation coatings like noble metals (Ag, Au)^8^, black compounds (ruthenium dioxides, rhenium trioxides)^9^, rare-earth hexaborides (lanthanum hexaborides)^10^,^11^, and semiconductor oxides (ITO, ATO, AZO, etc.)^12^,^13^,^14^, the NIR shielding ability and visible (Vis) transmittance of tungsten oxides can be remarkably superior due to the high free electron density, which can be utilized to produce localized surface plasma resonance^11^,^15^. Lately, our group have reported that cesium tungsten oxides (Cs_x_WO_3_) powders could be prepared by a water controlled-release solvothermal process with CsOH and WCl_6_ as raw materials, and it could exhibit excellent visible transparency and broad waveband absorption of 800 to 15,000 nm^16^,^17^. However, these raw materials are not environmentally friendly because of the easy hydrolysis of WCl_6_ and the volatilization of HCl^18^. To overcome this drawback, the one-dimensional potassium-doped tungsten bronze (K_x_WO_3_) powders were successfully synthesized with K_2_WO_4_ and K_2_SO_4_ through the hydrothermal process. The products could shield the NIR light with wavelength λ > 1000 nm, and this excellent heat shielding property implies that the K_x_WO_3_ might be the potential candidate for the smart window coated materials^19^.

If a smart window coating could be developed to not only shield the NIR but also degrade harmful pollutants, it would have a huge impact on deteriorating environmental issues and present self-cleaning effect simultaneously. Owing to photocatalytic and hydrophilic properties, semiconductor photocatalysts are widely and frequently employed to purify air and water contaminants^20^,^21^. Among various semiconductor photocatalysts, titanium dioxide (TiO_2_) is the most suitable photocatalyst for widespread environmental applications because of its long-term stability against photocorrosion, excellent photocatalytic activity and strong absorption of harmful ultraviolet (UV) light^22^,^23^,^24^,^25^,^26^. Due to the fact that a photocatalytic reaction occurs at the interface between catalyst surfaces and organic pollutants, it is highly feasible that the photocatalytic activity of TiO_2_ is strongly dependent on its surface properties^27^,^28^. Based on this speculation, surface-fluorinated TiO_2_ (F-TiO_2_) has been extensively investigated for its wonderful photocatalytic activity, which could be attributed to the enhanced generation of mobile free •OH radicals on the surface of F-TiO_2_^29^. Thus, F-TiO_2_ might be a promising photocatalyst to solve the environmental pollution concerns by relying on its highly efficient solar-light-driven photocatalytic activity^30^.

In our recent research, Cs_x_WO_3_/ZnO nanocomposite was prepared as a smart coating for photocatalytic environmental cleanup and heat insulation^31^. Despite numerous advantages of this film, the less environmental friendliness of the preparation process and the instability of ZnO are in urgent need to be solved^32^,^33^. In this work, a series of multifunctional NIR shielding-photocatalytic nanocomposite films were fabricated successfully through F-TiO_2_ and K_x_WO_3._ In these smart coatings, K_x_WO_3_ plays a part in shielding most of NIR light and holding high Vis light transparency, while F-TiO_2_ acts as both a photocatalyst to degrade harmful organic pollutants and a barrier to shield harmful ultraviolet light, which makes up the shortage of K_x_WO_3_ in the UV region. It’s worth mentioning that the K_x_WO_3_ nanorods were synthesized with Na_2_WO_4_ as tungsten source instead of K_2_WO_4_, which is considered as a lower-cost way than previously reported method of preparing tungsten bronze powders^19^,^34^. Furthermore, the heat-shielding and photocatalytic property of F-TiO_2_-K_x_WO_3_ nanocomposite films were evaluated and compared with that of ITO or P25 film to find out the optimal ratio of K_x_WO_3_ to F-TiO_2_ with a synergistic effect.

## Results and Discussion

**Figure 1:**
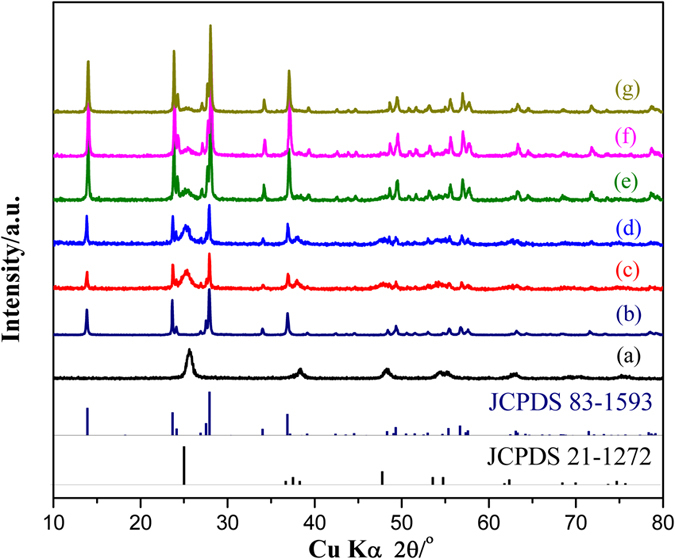
XRD patterns of pure (**a**) F-TiO_2_, (**b**) K_x_WO_3_ and different FT-KWO nanocomposites: (**c**) 3FT/KWO, (**d**) 2FT/KWO, (**e**) FT/KWO, (**f**) FT/2KWO, (**g**) FT/3KWO.

As shown in Fig. 1, XRD analysis has been employed for analyzing the crystalline phase of samples. The reflection in Fig. 1a matches best with the single anatase TiO_2_ (JCPDS 21-1272) phase, while the main peaks at 2θ values of 13.8^o^, 23.6^o^ and 27.8^o^ can be indexed respectively to (100), (002) and (200) crystal planes, which are readily indexed to the pure potassium tungsten bronze (K_0.26_WO_3_; JCPDS83-1593), as displayed in Fig. 1b. Furthermore, with the increasing content of F-TiO_2_, the intensities of the TiO_2_ peaks are increased obviously (Fig. 1c–g), revealing that FT-KWO nanocomposites are obtained successfully during the hydrothermal process. Meanwhile, peaks related to other phases are not observed in the synthesized samples, indicating that the F-TiO_2_ have not reacted with the K_x_WO_3_.

**Figure 2:**
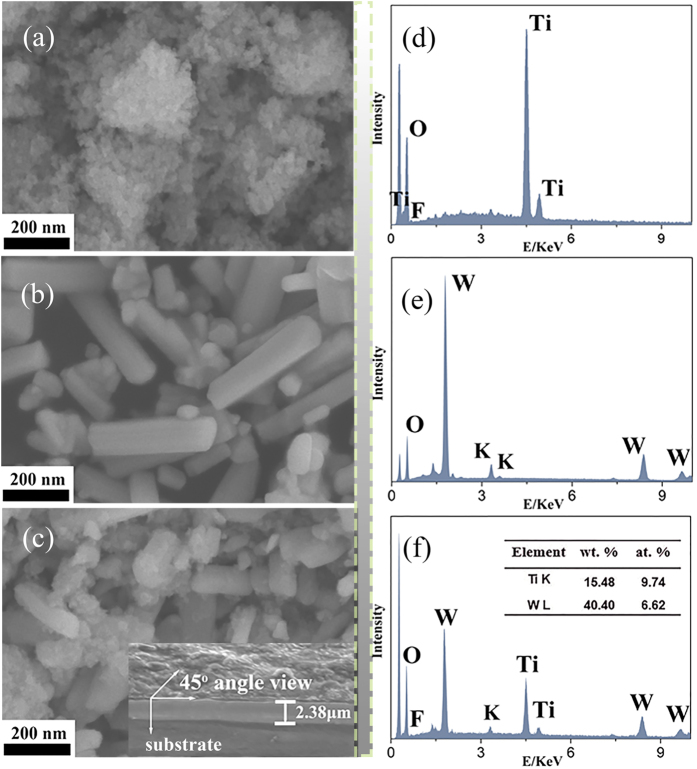
SEM images of (**a**) F-TiO_2_, (**b**) K_x_WO_3_, (**c**) FT/2KWO nanocomposites; EDX spectra of (**d**) F-TiO_2_, (**e**) K_x_WO_3_, (**f**) FT/2KWO nanocomposites. The insets in (**c**) shows SEM image of FT/2KWO films (at 45^o^ angle view).

The morphology of the as-prepared samples was characterized by SEM, which is shown in Fig. 2. As shown in Fig. 2a,b, the pure F-TiO_2_ is nanoparticles with relatively uniform size and the as-prepared K_x_WO_3_ powders exhibit smooth surface, regular nanorods with size of about 300–400 nm, which are corresponding to that of products synthesized with K_2_WO_4_ and K_2_SO_4_^19^. Figure 2c shows the SEM image of K_x_WO_3_ samples after the introduction of the F-TiO_2_, and it could be clearly seen that F-TiO_2_ particles are attached to the surface of K_x_WO_3_ nanorods, indicating the intimate contact between F-TiO_2_ and K_x_WO_3_. The inset in Fig. 2c shows the SEM image of FT/2KWO film at 45^o^, and the thickness of the film can be calculated to be about 3.37 μm, according to the equation: t = t’ × sin θ. Where t’ is the thickness shown in SEM image (t’ = 2.38 μm) and θ is the view angle (θ = 45^o^). In addition, EDX analysis confirms the distribution of Ti, O, F elements in F-TiO_2_ samples and K, W, O elements in K_x_WO_3_ samples as shown in Fig. 2d,e, respectively. As for FT/2KWO nanocomposites (Fig. 2f), it reveals the coexistence of Ti, O, F, K, and W elements as expected, which also indicates the existence of F-TiO_2_ and K_x_WO_3_ in FT/2KWO nanocomposites. Furthermore, according to EDX quantification (insert Fig. 2f), the atom percentages of Ti and W for the as-synthesized FT/2KWO nanocomposites are 9.74% and 6.62%, and the atom ratio of Ti to W is approximately 1.47, which is very close to the nominal value (Ti: W = 1.53). In addition, the Ti: W ratios are corresponding with the nominal ratios in all FT-KWO nanocomposites as displayed in Fig. S1, indicating that the elementary composition is well controlled by the experimental conditions.

**Figure 3:**
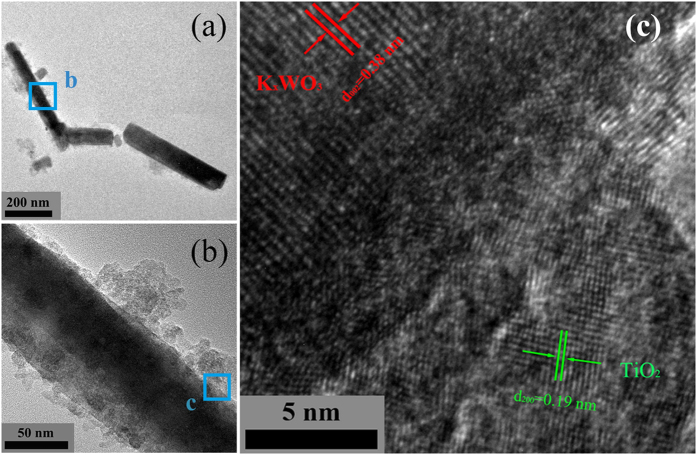
(**a**) Low-, (**b**) High-magnification TEM images and (**c**) HRTEM images of as-synthesized FT/2KWO nanocomposites.

To further obtain the microscopic morphology and structure information, the TEM and HRTEM analysis of as-synthesized FT/2KWO nanocomposites have been performed, as shown in Fig. 3. The TEM images of FT/2KWO nanocomposites (Fig. 3a,b) show the specific rod-like morphology with some nanoparticles attached, which is in accordance with SEM results. Moreover, The HRTEM image of the magnified view is given in Fig. 3c. The distance of 0.38 nm and 0.19 nm between the adjacent lattice fringes can be assigned to the (002) plane of hexagonal K_x_WO_3_ and the (200) plane of anatase TiO_2_ nanocrystals, respectively. Obviously, the FT/2KWO nanocomposites are formed with favourable nanosizd interfacial contact so as to exhibit multifunctional properties of NIR shielding and photocatalytic degradation.

The chemical composition of the FT/2KWO nanocomposite particles was also examined by XPS. The full range XPS spectra are presented in Fig. S2a. Peaks at binding energies corresponding to Ti, O, F, K, and W are clearly distinguished and no extra elements except carbon can be found in the spectra. The W4f core-level XPS spectra of the as-prepared FT/2KWO samples exhibit detailed information on the chemical state of core level tungsten, as shown in Fig. S2b. There are two spin-orbit doublets in this spectrum, which is attributed to W4f_7/2_ and W4f_5/2_: the peaks at 35.8 eV and 37.9 eV are attributed to W^6+^, while the peaks at 34.8 eV and 36.9 eV are assigned to W^5+^, which reaches a good agreement with the reported results. Meanwhile, the atomic contents of Ti and W in FT/2KWO were calculated from XPS and found to be 55.23 at. % and 36.21 at. %, corresponding to the nominal value (Ti: W = 1.53). It is suggested that the non-stoichiometric potassium tungsten bronzes are reduced compounds, and the co-existence of W^5+^ and W^6+^ is the necessary condition for the NIR shielding performance^31^.

**Figure 4:**
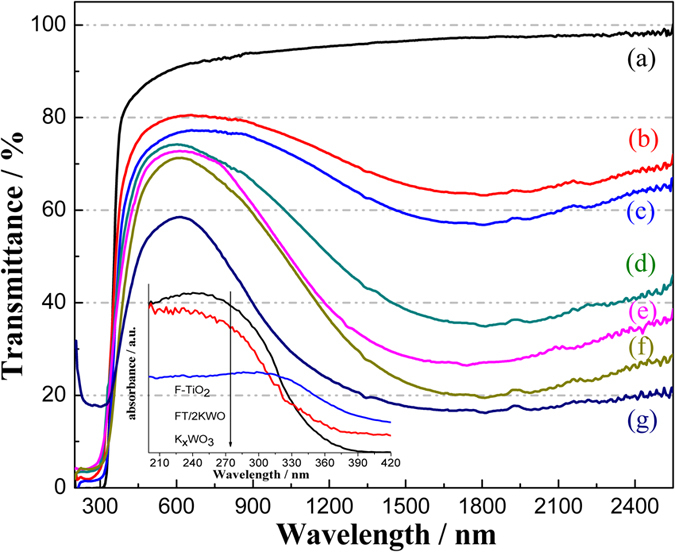
Transmittance spectra of (**a**) pure F-TiO_2_, different FT-KWO nanocomposites: (**b**) 3FT/KWO, (**c**) 2FT/KWO, (**d**) FT/KWO, (**e**) FT/2KWO, (**f**) FT/3KWO and (**g**) pure K_x_WO_3_ films. The inset shows the absorption spectra of different powders with the wavelength from 200 nm to 420 nm.

To study optical properties of the samples, the transmittance spectra of the as-prepared films are shown in Fig. 4. For the pure K_x_WO_3_ film (Fig. 4g), a great NIR shielding performance at the range of 780 to 2500 nm was observed, which could be closely related to the plasmon resonance of free electrons, interband transition and small polarons^35^. However, the visible light transparency and absorption of ultraviolet region (see inset in Fig. 4) are defective. On the contrary, these phenomena above are absent in the case of the film of pure TiO_2_ displayed in Fig. 4a, which exhibits no NIR shielding ability but excellent ultrviolet light absorption (see inset in Fig. 4) and high visible light transparency capability as a result of the narrow band gap of TiO_2_. As expected, all of the FT-KWO nanocomposite films retain all advantages of TiO_2_ and K_x_WO_3_ films in the range of UV, Vis and NIR. The inheritance of NIR absorption capability of composite could be attributed to the use of soft chemical method in synthesizing FT-KWO nanocomposite films in which the reduced W^5+^ ion was preserved. Specifically, with an increase in the K_x_WO_3_ content, the NIR shielding property becomes more pronounced while the visible light transmittance and UV shielding capability decreased. Even so, there will be a FT-KWO nanocomposite film with an appropriate mass ratio of F-TiO_2_ to K_x_WO_3_, exhibiting a great synergistic effect on blocking NIR and UV light as well as transmitting most of visible light.

**Figure 5:**
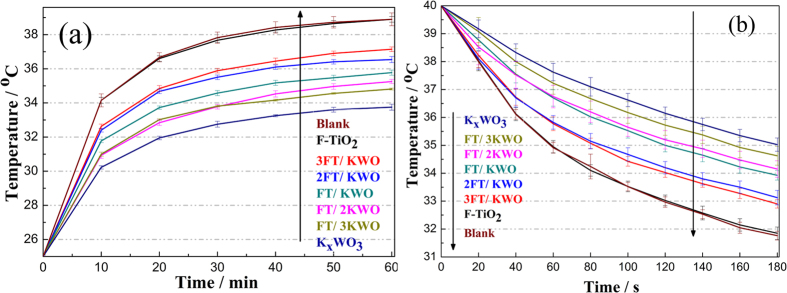
The inner temperature dependence on (**a**) irradiation time and (**b**) cooling time curves of sealed box covered with different films coated glass.

On the basis of the aforementioned optical properties of FT-KWO nanocomposite films, it is reasonable to suggest that these films show great potential for application as NIR shielding films. A thermal insulation experiment has been carried out in a sealed box. Figure 5 exhibits the inner temperature variation upon irradiation time and cooling time, and the temperature variations between the initial and final temperature are listed in Table S1. As shown in Fig. 5a, it’s obvious that the temperature increases significantly with irradiation time when the box is covered with blank or F-TiO_2_ coated glass, which proves that the F-TiO_2_ film has no NIR shielding capacity as displayed in Fig. 4a. Notably, the heating rates of glasses coated with FT-KWO nanocomposites are much lower than those of box covered with the blank or F-TiO_2_ coated glass, and the heating rate inside the box decreased distinctly with an increase in the K_x_WO_3_ content. For example, within 60 min irradiation, the temperature variation of box covered with 2FT/KWO nanoparticle film coated glass is 11.5 °C, while that of box covered with FT/2KWO film coated glass is depressed to 10.2 °C. In addition, the temperature variation curves with cooling time (Fig. 5b) show similar tendency as discussed above, that is to say, all of the FT-KWO nanocomposite films show good thermal insulation performance. Moreover, the temperature variation between the initial and final temperature slows down with the increasing content of K_x_WO_3_, as displayed in Table S1. The relatively slow heating rates during the heating and cooling time indicate the FT-KWO nanocomposite films have great thermal insulation capacity. The ITO glasses are widely used and well-known as effective NIR shielding material, as a control, the simulated experiment was carried out by irradiating the sealed box covered by ITO, FT + 2KWO (mechanically mix F-TiO_2_ and K_x_WO_3_), FT@2KWO (F-TiO_2_ film coating on the top of K_x_WO_3_ film) and 2KWO@FT (K_x_WO_3_ film coating on the top of F-TiO_2_ film) coated glass, respectively, and the temperature changes inside the boxes are plotted in Fig. S3a. By comparison, the box covered with the FT/2KWO film coated glass exhibited a slight lower temperature gradient than the box covered with other coated glasses both during the period of heating (Fig. S3a) and cooling (Fig. S3b). This phenomenon is corresponding to the transmittance spectra inserted in Fig. S3a. The results indicate superior thermal insulation capacity and potential practical application of the FT-KWO nanocomposite film.

**Figure 6:**
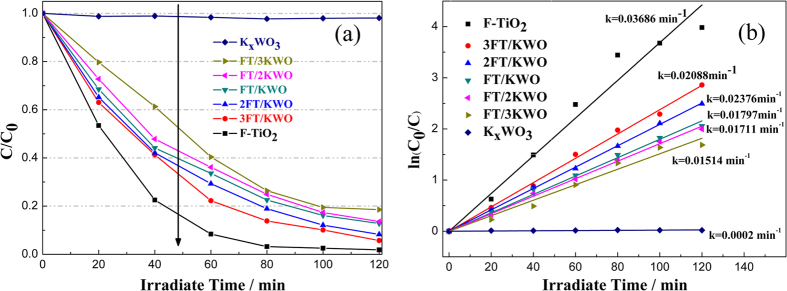
(**a**) Variation of MO concentration against irradiation time using F-TiO_2_ film, FT-KWO nanocomposite films with various K_x_WO_3_ contents and pure K_x_WO_3_ film under ultraviolet light irradiation and (**b**) plots of ln (C_0_/C) versus irradiation time for MO representing the fit using a pseudo-first-order reaction rate.

In order to confirm the photocatalytic activities of FT-KWO nanocomposite films, the degradation of MO by various films was carried out under the irradiation of UV light. As shown in Fig. 6a, F-TiO_2_ film exhibited high photocatalytic activity and 98% of the initial MO decreased after 80 min, whereas the K_x_WO_3_ film shows almost no effect on the degradation. Fortunately, these nanocomposite films are highly efficient for photocatalytic degradation of organic pollutant and the photocatalytic activity will be enhanced with the increase of F-TiO_2_ content in FT-KWO nanocomposites. For example, about 87% of the initial MO molecules are decomposed by the 3FT/KWO nanocomposite film, while in comparison, only 73% by the FT/3KWO film within 80 min. Based on previous studies, the degradation of dyes can be ascribed to a pseudo-first order reaction with a Langmuir–Hinshelwood model when the initial concentration of dye solution is small: ln (C_0_/C) = *k*t^36^. Where C is the concentration of MO after t min degradation, C_0_ is the initial concentration of MO, and *k* is the first-order reaction rate constant. Figure 6b exhibits the plots of ln (C_0_/C) versus irradiation time. Unusually, the FT/2KWO film exhibits similar photocatalytic activity to FT/KWO film where the *k* of FT/KWO is only about 1.05 times than that of FT/2KWO. Meanwhile, considering the temperature variation curves displayed in Fig. 5, the thermal insulation capacity of FT/2KWO film has gained a noticeable improvement than that of FT/KWO film. In other words, the FT-KWO film shows the best multifunctional properties when the mass ratio of F-TiO_2_ to K_x_WO_3_ is 1:2. To further investigate the advantages of FT-KWO nanocomposite film, as a comparison, the photocatalytic activities of P25, FT + 2KWO, FT@2KWO, 2KWO@FT and F-TiO_2_’ (where the F-TiO_2_ content in this film is equal to that of FT/2KWO film) films were measured under the same condition. As displayed in Fig. S4, the photocatalytic activity sequence of these films is FT/2KWO ~ P25 > FT + 2KWO > F-TiO_2_’ ~ FT@2KWO > 2KWO@FT and the enhancement of the photocatalytic activity could be attributed to the close interfacial contact and strong interaction between F-TiO_2_ and K_x_WO_3_ in FT/2KWO film. Besides, the introduction of K_x_WO_3_ with excellent electronic conductivity can promote the photogenerated electron transport to the surface of the composite more easily, thus inhibiting the recombination between photogenerated electrons and holes^34^,^37^,^38^. However, when it comes to FT + 2KWO film and others, the F-TiO_2_ and K_x_WO_3_ are independent (see SEM image inset Fig. S4) to generate nearly no synergistic effect. It is abnormal that only 53% of the initial MO molecules are decomposed by 2KWO@FT nanocomposite film within 120 min, which might be attributed to the covered surface of F-TiO_2_ limits the photocatalytic reaction to some extent^39^. In conclusion, these results mentioned above further confirm that the FT/2KWO nanocomposite film is effective for photocatalytic degradation of organic pollutant and very promising for practical application in smart window.

In consideration of the practical application of these nanocomposite films, the photocatalytic stability of the as-prepared FT/2KWO nanocomposite film was further investigated by the cycle experiment. As shown in Fig. S5a, the photocatalytic activity of the FT/2KWO nanocomposite film remains still unreduced after four consecutive cycles, thus indicating the good stability of the film during the photocatalytic experiment. Furthermore, the thermal insulation capacity of the FT/2KWO film after the fourth photocatalytic experiment was investigated and exhibited in Fig. S5b. It is clear that the temperature variation of box is about 10.5 °C (Fig. S5b-2) when it was covered with the FT/2KWO film coated glass after the fourth photocatalytic experiment, which is slightly higher than that of new FT/2KWO film coated glass (Fig. S5b-3), but much lower than that of blank glass (Fig. S5b-1). It thus indicates the great reusability of the FT-KWO nanocomposite films.

**Figure 7:**
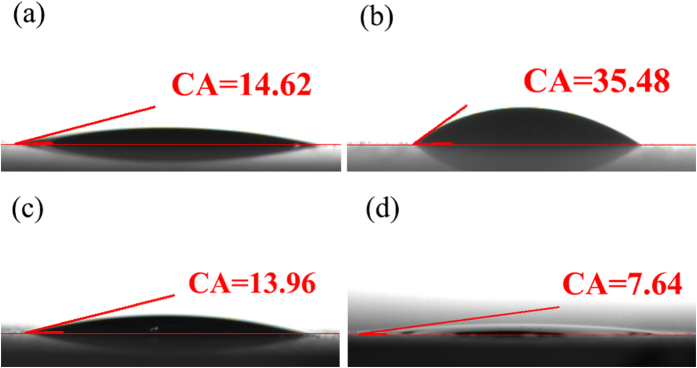
Contact angle of a water drop in air on new (**a**) F-TiO_2_, (**b**) K_x_WO_3_, (**c**) FT/2KWO films and (**d**) FT/2KWO film after the fourth photocatalytic experiment.

To identify the surface hydrophilic properties of the nanocomposite films, the contact angle (CA) value of the as-prepared FT/2KWO film was measured and compared with that of pure TiO_2_, K_x_WO_3_ film, as shown in Fig. 7. Notably, the CA value of K_x_WO_3_ film (CA = 35.48) is significantly higher than that of F-TiO_2_ film (CA = 14.62), which indicates the F-TiO_2_ film has good hydrophilic property. Meanwhile, the CA value of FT/2KWO film (CA = 13.96) has not been affected by the introduction of K_x_WO_3_ and the similar CA values between F-TiO_2_ film and FT/2KWO film reveal the hydrophilic nature of prepared FT/2KWO film. Interestingly, the CA value of FT/2KWO film decreased to 7.64^°^ after the fourth photocatalytic experiment, as displayed in Fig. 7d. This phenomenon could be attributed to the photoinduced hydrophilicity caused by reconstruction of surface hydroxyl groups^40^,^41^. The above results confirm the FT/2KWO film is advantageous for applications in antifogging and self-cleaning coatings.

**Figure 8:**
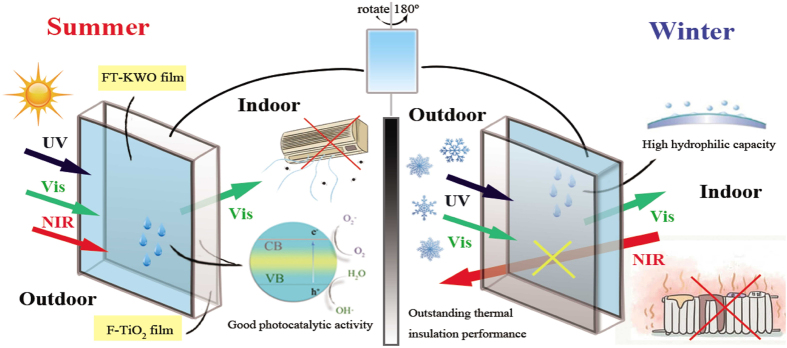
The working model of the FT-KWO and F-TiO_2_ coated window applied to different conditions.

A working model illustrating the multifunctionality of the FT-KWO and F-TiO_2_ coated window is summarized in Fig. 8. Obviously, this smart window plays different roles in various conditions. In summer days, the FT-KWO film side towards the outside; with the irradiation of the solar light, the smart window can not only block most of the NIR lights for heat preservation and keep cool indoor, but also isolate harmful UV light and transmit Vis light. In winter days, this window should be rotated 180° to make the FT-KWO film side towards the inside. In this case, the smart window not only reduces heat loss from inside to outside, but also blocks UV light and transmits Vis light effectively. Meanwhile, the high hydrophilic capacity can control the production of water vapour on the window and enhance the visibility availably. To sum up, this smart window will minimize the usage of air conditioning and heaters to control the redundant electricity consumption and promote the solution of energy crisis. In addition, both sides will absorb UV light to motivate photocatalytic reaction owing to the existence of F-TiO_2_, so as to degrade the harmful organic air pollutants and help solve the environmental issues subtly.

In summary, the F-TiO_2_-K_x_WO_3_ multifunctional nanocomposites have been fabricated successfully through a low-cost and eco-friendly method. Moreover, the FT-KWO films exhibited excellent multifunctional performance of NIR, UV light insulation, Vis transparency and photocatalytic activity. A competitive relation was observed between F-TiO_2_ and K_x_WO_3_: the photocatalytic activity will be decreased while the thermal insulation performance improved with the increase of K_x_WO_3_ content in FT-KWO nanocomposite films. As the optimal proportion of K_x_WO_3_ to F-TiO_2_, the FT/2KWO nanocomposite film shows a significant multifunctional property of outstanding NIR, UV light shielding performance, high photocatalytic activity on degradation of harmful organic pollutants and excellent hydrophilic capacity. It is worth mentioning that its thermal insulation capacity is better than that of ITO, while the photocatalytic activity is surpassing P25. Therefore, FT-KWO nanocomposite film could have great potential applications as smart windows coating material, so as to help solve the energy crisis and deteriorating environmental issues in a convenient way.

## Methods

### Preparation of K_x_WO_3_ nanorods

K_x_WO_3_ nanorods were synthesized on the basis of a procedure reported previously. All reagents were of analytical grade and used without further retreatment. Specifically, 1.6493 g Na_2_WO_4_·2H_2_O and 1.7424g K_2_SO_4_ were dissolved in 50 ml deionized water under magnetic stirring followed by adding 3 mol/L HCl to adjust pH to 1.5. After that, the resultant solution was transferred into a dried Teflon-lined autoclave with 100 mL internal volume and kept it at 200 °C for 24 h. After natural cooling to room temperature, the intermediate products were obtained after being washed with water and ethanol for three times respectively and dried at 50 °C overnight. Finally, a certain amount of as-prepared samples was reduced in H_2_ (5 vol %)/N_2_ atmosphere at 500 °C for 1 h to obtain the potassium tungsten bronze.

### Preparation of FT-KWO nanocomposites

The as-prepared K_x_WO_3_ nanorods were redispersed via the assist of ultrasonication in 30 mL deionized water to obtain the homogeneous suspension. Then, 0.0713g NH_4_HF_2_ and 7.5 ml absolute ethyl alcohol with 1.71 ml tetrabutyl titanate (TBOT) were added dropwise to the suspensions under vigorous stirring for 30 min. Finally, the above suspensions were transferred into 50 mL Teflon-lined autoclaves and maintained at 150 °C for 10 h. After this hydrothermal reaction, the suspensions were centrifuged at 17000 rpm followed by washing with distilled water and ethanol twice, and then dried in an oven at 80 °C for 2 h. The nominal contents of K_x_WO_3_ additives were 25 wt.%, 33 wt.%, 50 wt.%, 67 wt.% and 75 wt.%, respectively for the FT-KWO nanocomposites. These samples are labelled as 3FT (F-TiO_2_)/KWO (K_x_WO_3_), 2FT/KWO, FT/KWO, FT/2KWO, and FT/3KWO, respectively. For comparison, pure F-TiO_2_ and K_x_WO_3_ were synthesized under the same condition.

### Preparation of FT-KWO nanocomposite films

The NIR shielding and photocatalysis properties of F-TiO_2_-K_x_WO_3_ nanocomposites were evaluated by coating them onto quartz glass substrates (4 cm*4 cm*1 mm). In a typical film synthesis process, 0.2 g samples were dispersed into a mixed solution with 1.24 g collodion and 1.33 g absolute ethyl alcohol under magnetically stirring, thus forming homogeneous colloidal dispersions. Subsequently, the above slurries were spin-coated on the quartz glass substrates at 2500 rpm for 60 s, rinsed with absolute ethyl alcohol and dried at 60 °C for 30 min. For comparison, the P25, ITO and blank films were prepared under the identical conditions. In addition, the synthesis process of other films was presented in the supporting information.

### Characterization

The phase purity of samples was analyzed by X-ray powder diffraction (XRD) using a Bruker D2 PHASER X-ray diffractometer with graphite monochromator using Cu Kα radiation (λ = 1.54184 Å) at room temperature. The morphology of the sample and the energy dispersive X-ray spectroscopy (EDS) spectrum were detected by field emission scanning electron microscopy (FESEM, Hitachi, 30 S-4800). Moreover, the transmittance of films was measured with a Perkin Elmer 950 spectrometer. Besides, transmission electron microscopy (TEM) and high-resolution transmission electron microscopy (HRTEM) images were collected on an F30 S-TWIN electron microscope (Tecnai G2, FEI Company). X-ray photoelectron spectroscopy (XPS, PHI-5702, Physical Electronics) was performed using a monochromated Al Ka irradiation. The chamber pressure was ~3 × 10^−8^ Torr under testing conditions. The surface hydrophilic properties of the as-prepared films were characterized by detecting the water contact angle measured by a contact angle meter (JC2000C) with 4 μL water droplet under ambient conditions.

### Evaluation of NIR shielding property

To evaluate the thermal insulation properties of FT-KWO nanocomposite films, a simulated room was built with a sealed plastic box (11 cm*11 cm*11 cm) covered with different films coated glasses under the irradiation of a 275 W infrared lamp. The experiments were carried out at the room temperature of 25 °C and temperature variation was monitored by a thermodetector with four thermocouples every ten minutes. Meanwhile, the heat preservation performance of these films was evaluated in the same plastic box without the irradiation of infrared lamp, but the glasses were rotated 180° to make the FT-KWO film side toward the inside with the initial temperature being 40 °C.

### Evaluation of photocatalytic activity

The photocatalytic activities of the FT-KWO nanocomposite films were evaluated by measuring the degradation ratio of methyl orange (MO). The initial concentration of MO solution was 10 mg/L^−1^, and the quartz glass substrate coated with FT-KWO nanocomposite film was immersed in 5 mL MO solution. A 500 W high pressure Hg lamp was employed for the ultraviolet irradiation source and positioned 10 cm away from the reactor to trigger the photocatalytic reaction. In addition, a certain volume of MO solution was withdrawn at selected times and analysed by measuring the light absorption of the clear solution at 464 nm (λ_max_ for MO solution) using a spectrophotometer (LG-722SP).

## Additional Information

**How to cite this article**: Liu, T. *et al.* Smart window coating based on F-TiO_2_-K_x_WO_3_ nanocomposites with heat shielding, ultraviolet isolating, hydrophilic and photocatalytic performance. *Sci. Rep.* **6**, 27373; doi: 10.1038/srep27373 (2016).

## Supplementary Material

Supplementary Information
