# Supplementary material for: Smart window coating based on F-TiO2-KxWO3 nanocomposites with heat shielding, ultraviolet isolating, hydrophilic and photocatalytic performance
Source: Sci Rep. 2016 Jun 6;6:27373. doi: 10.1038/srep27373 (PMC4893629; doi:10.1038/srep27373)
Supplement: Supplementary Information [file srep27373-s1.doc]

**Smart window coating based on** **F-TiO_2_-K_x_WO_3_­ nanocomposites with heat shielding, ultraviolet isolating, hydrophilic and photocatalytic performance**

**Tongyao Liu^1^, Bin Liu**^1^,**^*^, Jing Wang**^1^**, Linfen Yang**^1^**, Xinlong Ma**^1^**, Hao Li**^1^**, Yihong Zhang^1^, Shu Yin^2^, Tsugio Sato^2^, Tohru Sekino^3^ & Yuhua Wang**^1^,**^*^**

^1^ Department of Materials Science, School of Physical Science and Technology, Lanzhou University, Lanzhou, 730000, China

^2^ Institute of Multidisciplinary Research for Advanced Materials, Tohoku University, 2-1-1 Katahira, Aoba-ku, Sendai, Japan

^3^ The Institute of Scientific and Industrial Research, Osaka University, Japan

^*^ Corresponding author: Yuhua Wang, Ph.D, Professor; Bin Liu, Ph.D.

Tel: +86-931-8912772; Fax: +86-931-8913554

E-mail address: [wyh@lzu.edu.cn;](mailto:wyh@lzu.edu.cn;) liubin@lzu.edu.cn.

**Supplementary Information**

**Preparation of FT@2KWO, 2KWO@FT and FT+2KWO films**

Specifically, the preparation of FT@2KWO film is as follows: 0.1333g K_x_WO_3_ was dispersed into a mixed solution with 1.24g collodion and 1.33g absolute ethyl alcohol under magnetically stirring to form homogeneous colloidal dispersions named A, 0.0667g F-TiO_2_ was dispersed in the same solution to form the colloidal dispersions named B. Subsequently, a certain amount of A was spin-coated on the quartz glass substrate at 2500 rpm for 60s and annealed at 60 °C for 30 min. Then, the same amount of B was spin-coated on the top surface of the FT film to form the double-layer film. Analogously, 2KWO@FT film was prepared with B (F-TiO_2_ slurry) and A (K_x_WO_3_ slurry) in a same method. As for the preparation of FT+2KWO film, 0.0667g F-TiO_2_ and 0.1333g K_x_WO_3_ were mixed mechanically and spin-coated in a similar process.


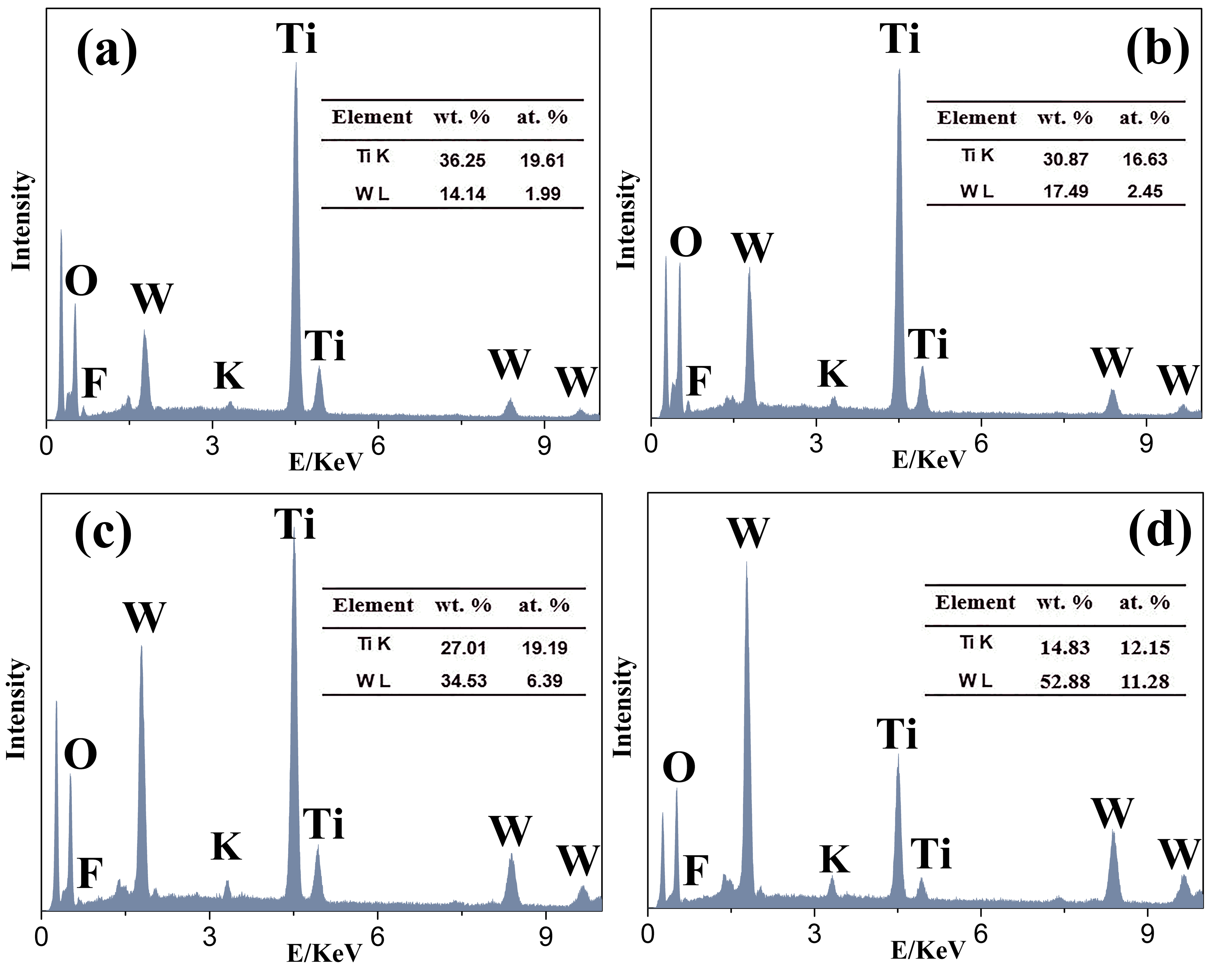


**Figure S1.** EDX spectra of (a) 3FT/KWO, (b) 2FT/KWO, (c) FT/KWO and (d) FT/3KWO nanocomposites. The tables inserted depict the compositions of nanocomposites.


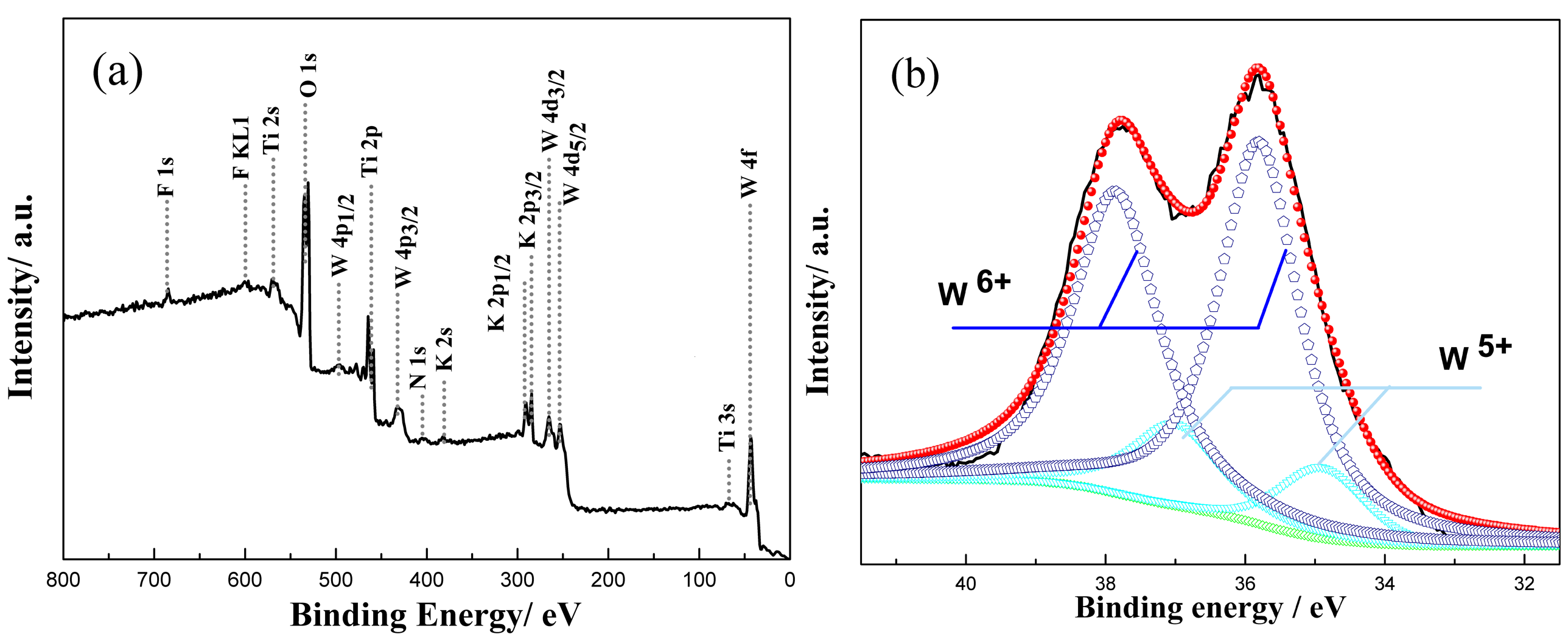


**Figure S2.** (a) Full range and (b) W4f core-level XPS spectra of the as-prepared FT/2KWO nanocomposites.


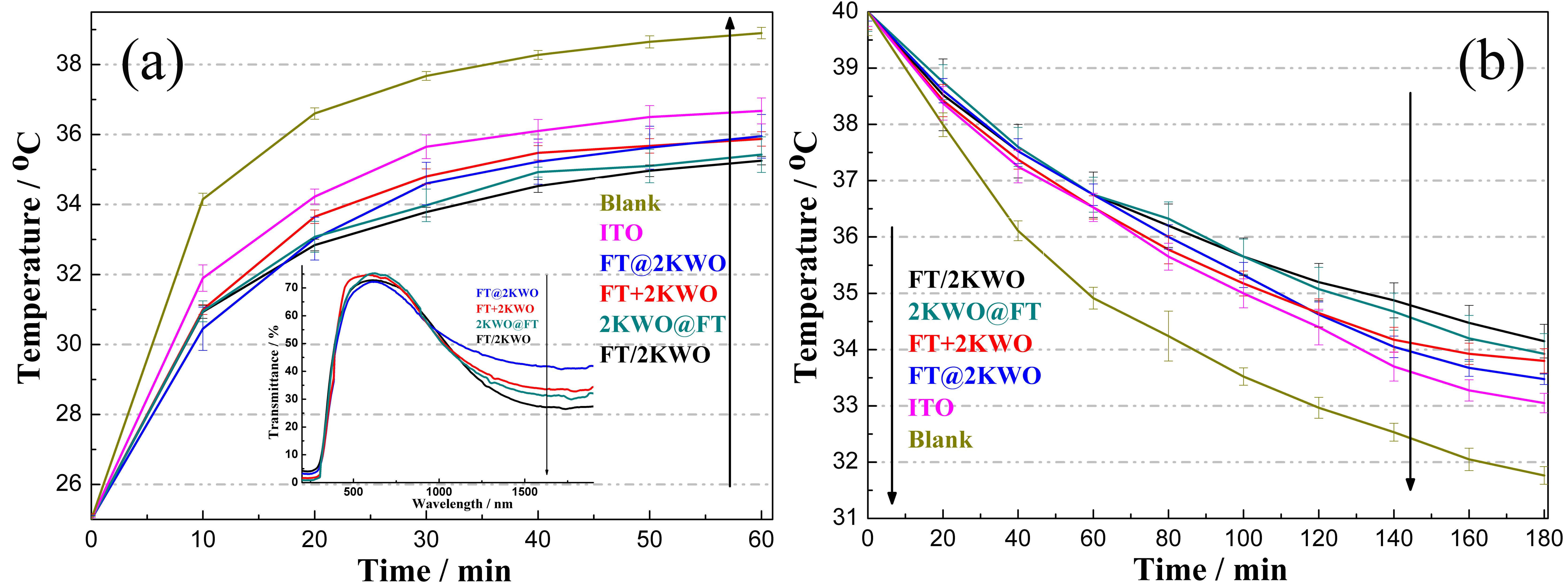


**Figure S3.** The inner temperature dependence on (a) irradiation time and (b) cooling time curves of sealed box covered with different films coated glass. (The inset in FigureS3a shows transmittance spectra of FT/2KWO, 2KWO@FT, FT+2KWO and FT@2KWO films)


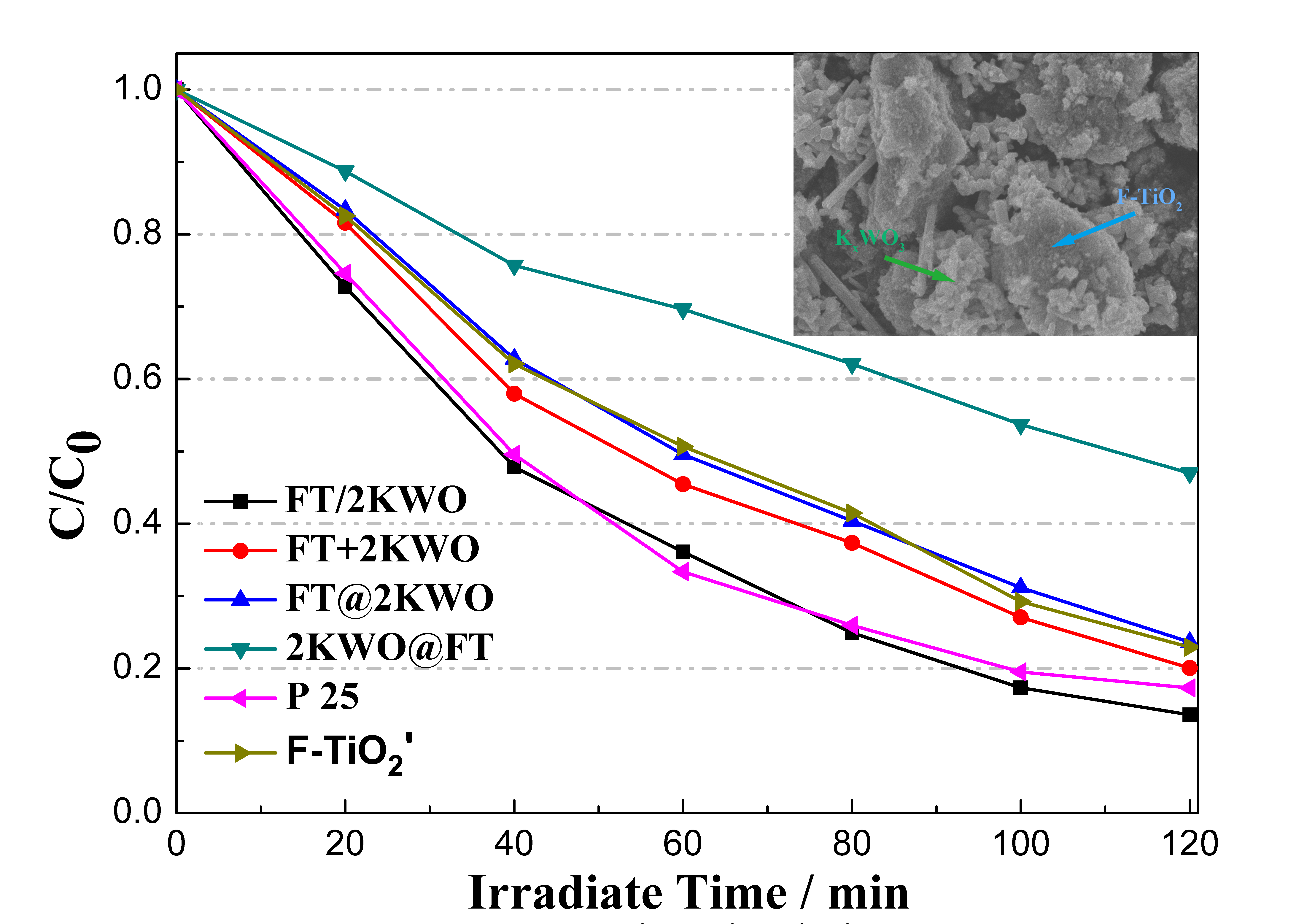


**Figure S4.** Variation of MO concentration against irradiation time using different films under ultraviolet light irradiation. (The inset shows SEM image of FT+2KWO powders)





**Figure S5.** (a) Repeated photocatalytic degradation of MO solution in the presence of the FT/2KWO nanocomposite film and (b) the inner temperature curves of sealed box covered with (1) blank glass, (2) FT/2KWO film after the fourth photocatalytic experiment and (3) new FT/2KWO film coated glasses.

**Table S1.** The temperature variation (ΔT) between the initial and final temperature with the existence of different film coated glasses

| Film  ΔT | Blank | F-TiO_2_ | 3FT/KWO | 2FT/KWO | FT/KWO | FT/2KWO | FT/3KWO | K_x_WO_3_ |
| --- | --- | --- | --- | --- | --- | --- | --- | --- |
| ΔT_1_^a^ (^o^C ) | 13.9 | 13.9 | 12.1 | 11.5 | 10.7 | 10.2 | 9.8 | 8.7 |
| ΔT_2_^b^ (^o^C ) | 8.3 | 8.2 | 7.1 | 6.9 | 6.1 | 5.9 | 5.4 | 5.0 |
| ^a^ The temperature variation with the irradiation of infrared lamp. ^b^ The temperature variation during the cooling time. | | | | | | | | |
